# Influence of Preconception Maternal BMI and Gestational Weight Gain on Perinatal Outcomes

**DOI:** 10.7759/cureus.110194

**Published:** 2026-06-03

**Authors:** Sunita Palo, Sabina Terzic

**Affiliations:** 1 Neonatal Intensive Care Unit, Clinical Center University of Sarajevo, Sarajevo, BIH

**Keywords:** body mass index, gestational weight gain, neonatology, perinatal outcomes, perinatology

## Abstract

Introduction

Inadequate BMI in women before conception, as well as non-optimal gestational weight gain (GWG) during pregnancy, affects the health of the mother and newborn. The aim of this study was to examine the influence of maternal BMI before conception and GWG during pregnancy on perinatal outcomes in newborns and mothers.

Methods

A retrospective study was conducted at the Clinic for Gynecology and Obstetrics and the Pediatric Clinic, Clinical Center University of Sarajevo (CCUS), from January 1, 2024 to December 31, 2024. A total of 2,067 participants were included in the study. Descriptive statistical methods were used. The analyzed perinatal outcomes included method of conception, type of delivery, birth weight of newborns, Apgar score at 1 minute, need for NICU hospitalization, and development of disease or other complications related to maternal health.

Results

The majority of the participants, 60.9% (n = 1,259), had a normal BMI, 27.1% (n = 559) were overweight, 9.1% (n = 189) were obese, and 2.9% (n = 60) were underweight. The average GWG was 15.1 kg; 46.0% (n = 952) of the subjects had excessive GWG, 37.4% (n = 772) had GWG within the recommended range, and 16.6% (n = 343) had low GWG. A statistically significant association was found between maternal BMI and mode of conception, type of delivery, prematurity, birth weight, Apgar score, need for NICU hospitalization, and presence of maternal diseases. GWG was significantly associated with infant birth weight, Apgar score, and the presence of maternal diseases.

Conclusion

The conducted study provides data on the significant and non-negligible association of inadequate maternal BMI before pregnancy and non-optimal GWG during pregnancy with adverse perinatal outcomes. Education and preventive promotion of body weight control must become a priority in public health among women and future mothers to reduce the risk of less favorable perinatal outcomes for the mother and newborn.

## Introduction

Overweight and obesity represent one of the greatest global public health challenges of the 21st century, as obesity has reached epidemic proportions worldwide. In 2022, 43% of adults aged 18 years and older were overweight, and 16% were living with obesity [1,2]. Maintaining a healthy BMI is essential for optimizing fertility. Both undernutrition and overweight pose challenges that can make conception more difficult, reduce fertility, and increase the time needed for conception. Obesity-related comorbidities increase the risk of adverse outcomes for both mother and child [3].

BMI is a simple indicator of body fat based on height and weight. It is a quick, safe, and reliable screening measure to assess a person’s weight in relation to their height. Although BMI does not directly measure body fat, it is related to other measures that include the amount, location, and distribution of body fat [1,4]. The advantages of BMI compared to direct measurements of body fat are that it is quick and easy to calculate, inexpensive, and non-invasive.

The WHO defines the normal BMI range as 18.5-24.9 kg/m² [1,4]. Key categories include underweight (BMI <18.5), normal weight (BMI 18.5-24.9), overweight (BMI 25.0-29.9), and obesity (BMI ≥30.0) [1,4]. The total gestational weight gain (GWG) of a pregnant woman is the result of several factors, such as the mother’s diet, fetal growth, the amount of amniotic fluid, maternal fat stores, and fluid retention. How much weight a pregnant woman should gain during pregnancy is based on her pre-pregnancy BMI. Based on the Institute of Medicine (IOM) guidelines, for a woman of normal body weight (BMI 18.5-24.9), the recommended GWG is 11.5-16 kg. Weight gain generally follows a pattern of 1-2 kg in the first trimester of pregnancy, and then approximately 0.5 kg per week for the rest of the pregnancy [5,6]. Recommended GWG according to BMI for a singleton pregnancy is as follows: underweight (BMI <18.5): 13-18 kg; normal weight (BMI 18.5-24.9): 11.5-16 kg; overweight (BMI 25.0-29.9): 7-11.5 kg; and obesity (BMI ≥30.0): 5-9 kg [5,6]. For multiple pregnancy, the recommended GWG is as follows: underweight (BMI <18.5): 23-28 kg; normal weight (BMI 18.5-24.9): 17-25 kg; overweight (BMI 25.0-29.9): 14-23 kg; and obesity (BMI ≥30.0): 11-19 kg [5,6].

Undernutrition and overweight in pregnant women, as well as non-optimal GWG, affect maternal and newborn health. The aim of this study is to examine the impact of maternal preconception BMI and GWG on perinatal outcomes in newborns and mothers.

## Materials and methods

This is a retrospective study conducted at the Clinic for Gynecology and Obstetrics and the Pediatric Clinic, Clinical Center University of Sarajevo (CCUS), from January 1, 2024, to December 31, 2024. The study included a total of 2,067 subjects. Subjects for whom all necessary data were incomplete were excluded. The data were anonymized and obtained from medical records. The authors received approval for the study from the Ethics Committee of CCUS, approval number 51-04-9-22095/23, dated June 6, 2023.

The mother's BMI before pregnancy was calculated according to the standard formula (kg/m²) and categorized as follows: underweight (<18.5 kg/m²), normal body weight (18.5-24.9 kg/m²), overweight (25.0-29.9 kg/m²), and obese (≥30.0 kg/m²). The mother's weight before pregnancy was self-reported. GWG was classified as too low, within the recommended limits, or too high based on the IOM recommendations, according to the mother's BMI before pregnancy and gestational age at birth, using weekly weight gain rates.

The analyzed perinatal outcomes included mode of conception, type of delivery, birth weight of newborns, Apgar score at 1 minute, need for NICU hospitalization, and development of diseases and other complications related to maternal health.

Descriptive statistical methods were used. The chi-square test was used to analyze associations between categorical variables, the Kruskal-Wallis test was used to compare continuous variables among multiple groups, and the Wilcoxon post-hoc test with Bonferroni correction was applied. Fisher's exact test was used for categorical comparisons with small expected cell frequencies, specifically for the analyses of maternal BMI category with NICU hospitalization, maternal BMI category with in vitro fertilization (IVF) pregnancy, and GWG category with IVF pregnancy. A p-value <0.05 was considered statistically significant. Nonparametric and exact tests were chosen because the data were heterogeneous, partially non-normally distributed, and had uneven frequency distributions, making these tests the most appropriate for this type of clinical database.

## Results

There were 2,263 singleton births at the Clinic for Gynecology and Obstetrics, CCUS, during 2024, of which 2,067 were included in our study according to the previously defined criteria. The average age of the mothers was 31.4 years. The median BMI of mothers before pregnancy was 23.6 kg/m². The largest proportion of respondents had a normal BMI, 60.9% (n = 1,259), while 27.1% (n = 559) were overweight and 9.1% (n = 189) were obese. Underweight mothers accounted for 2.9% (n = 60). The average GWG was 15.1 kg. The largest proportion of mothers had excessive GWG, 46.0% (n = 952); 37.4% (n = 772) had GWG within the recommended range, while 16.6% (n = 343) had inadequate GWG, below the recommended range. Prematurity was recorded in 16.0% (n = 331) of cases. Of the total number of births analyzed, 35.3% (n = 729) were completed by cesarean section. Conception through IVF was recorded in 3.6% (n = 75) of cases. The basic characteristics of the mothers and newborns are described in Table 1.

**Table 1:** Baseline characteristics of the mothers and newborns. IVF: In vitro fertilization.

Variable	Value
Maternal age, years	31.4 ± 6.0 (median 31; range 15-47)
Pre-pregnancy BMI, kg/m²	24.3 ± 4.6 (median 23.6; range 16.0-40.5)
Maternal BMI categories, n (%)	
Underweight	60 (2.9%)
Normal weight	1,259 (60.9%)
Overweight	559 (27.1%)
Obese	189 (9.1%)
Gestational weight gain, kg	15.1 ± 6.9 (median 15.1; range 10-34)
Gestational weight gain categories, n (%)	
Inadequate	343 (16.6%)
Recommended	772 (37.4%)
Excessive	952 (46.0%)
Prematurity (<37 weeks of gestation)	331 (16.0%)
Cesarean section	729 (35.3%)
IVF conception	75 (3.6%)

Statistically significant associations were found between maternal BMI before pregnancy and type of delivery, prematurity, birth weight, Apgar score, need for NICU hospitalization, presence of maternal disease, and mode of conception. Maternal BMI before pregnancy showed a strong and consistent association with all analyzed perinatal outcomes.

GWG was significantly associated with newborn birth weight, Apgar score, and presence of maternal diseases. Table 2 shows the association between maternal BMI, GWG, and perinatal outcomes.

**Table 2:** Associations between maternal BMI, gestational weight gain, and perinatal outcomes. IVF: In vitro fertilization; GWG: Gestational weight gain.

Variable	Test statistic	p-value
Maternal BMI × type of delivery	χ² = 18.05	<0.001
Maternal BMI × prematurity	χ² = 13.94	0.003
Maternal BMI × birth weight	χ² = 63.44	<0.001
Maternal BMI × Apgar score at 1 minute	χ² = 34.10	<0.001
Maternal BMI × NICU hospitalization	-	0.048
Maternal BMI × maternal disease	χ² = 92.11	<0.001
Maternal BMI × IVF conception	-	<0.001
GWG × type of delivery	χ² = 1.32	0.52
GWG × prematurity	χ² = 1.15	0.56
GWG × birth weight	χ² = 86.21	<0.001
GWG × Apgar score at 1 minute	χ² = 19.47	<0.001
GWG × NICU hospitalization	χ² = 3.80	0.15
GWG × maternal disease	χ² = 31.93	<0.001
GWG × IVF conception	-	0.058

A statistically significant association was found between maternal BMI categories and type of delivery (χ² = 18.05; p < 0.001). The incidence of cesarean section was higher in overweight and obese mothers compared to mothers with normal BMI. Underweight mothers had a higher incidence of vaginal delivery.

The analysis showed a statistically significant association between maternal BMI category before conception and the occurrence of prematurity (χ² = 13.94; p = 0.003). Prematurity was more common in overweight and obese mothers.

**Figure 1:**
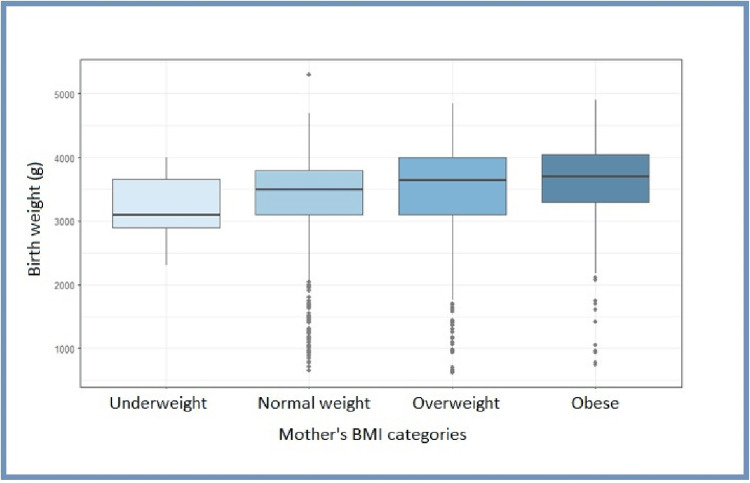
Birth weight of newborns according to maternal BMI category.

A highly statistically significant difference was found in the birth weight of newborns between maternal BMI categories (Kruskal-Wallis χ² = 63.44; p < 0.001). Figure 1 shows that newborns of mothers with a higher BMI had higher birth weight, and newborns of underweight mothers had lower birth weight. Post-hoc analysis showed significant differences between almost all BMI categories.

We also found an association between maternal BMI categories and the presence of diseases or complications during pregnancy (χ² = 92.11; p < 0.001). The most common maternal diseases were gestational hypertension, gestational diabetes mellitus, preeclampsia, and postpartum hemorrhage. The frequency of diseases increased with increasing maternal BMI. A statistically significant association was also found between GWG categories and the presence of maternal diseases (χ² = 31.93; p < 0.001). Mothers with excessive GWG had a higher frequency of diseases compared to other categories of body weight gain.

## Discussion

In our one-year study, we included 2,067 parturient women with singleton pregnancies. The majority had a normal BMI, 60.9% (n = 1,259), while 27.1% (n = 559) were overweight, 9.1% (n = 189) were obese, and 2.9% (n = 60) were underweight. In the study by Gandhi S et al., 49.2% of respondents had a normal BMI, 28.4% were underweight, 15.6% were overweight, and 6.8% were obese [7]. In a large study by Liu P et al., 58.03% of subjects had a normal BMI, 22.08% were overweight, 11.7% were obese, and 8.18% were underweight, which is similar to our results [8]. We note that a significantly lower percentage of underweight mothers was included in our research compared to similar studies [7,8].

In our study, 37.4% (n = 772) of the subjects had GWG within the normal recommendations, 16.6% (n = 343) had inadequate GWG, and 46.0% (n = 952) had excessive GWG. In a large cohort study by Liu X et al., 24.5% of subjects had GWG below the recommended range, 27.6% had GWG within the guidelines, and 47.9% had GWG greater than recommended [9]. We note that in our study, more mothers had normal GWG. In the study by Nepal J et al., 52.4% of participants had excessive GWG, which is similar to our results [10].

According to the WHO, the use of cesarean section continues to increase globally, now accounting for 21% of all deliveries, with large differences between regions. It is estimated that by 2030, almost one-third (29%) of all deliveries will be by cesarean section [11,12]. In our study, 35.27% (n = 729) of deliveries were completed by cesarean section. The high percentage can be explained by the fact that the CCUS is a tertiary-level hospital, where the most complicated pregnancies from a region with a population of about 1 million are managed, which is also described in the study by Nepal J et al., who reported a cesarean section rate of up to 45% [10].

In our study, a statistically significant association was found between maternal BMI categories and type of delivery (χ² = 18.05; p < 0.001). The incidence of cesarean section was higher in overweight and obese mothers compared to mothers with normal initial BMI. A large systematic meta-analysis by Zhang Y et al. also demonstrated that the incidence of cesarean section was statistically significantly higher in overweight mothers [13]. Similar results were also found in the studies by Gandhi S et al. and Szablewska A et al. [7,14].

Our study did not show a significant association between GWG and type of delivery (χ² = 1.32; p = 0.52). These results are not consistent with those of the studies by McElfish PA et al. and Gearhart R et al., which showed a statistically significantly higher incidence of cesarean section in women with high GWG [15,16]. In their research, Nepal J et al. did not demonstrate a statistically significant association between maternal weight gain during pregnancy and the frequency of cesarean delivery, which is in accordance with our results [10].

Our research showed that the occurrence of prematurity was statistically significantly more frequent in mothers with high BMI, but there was no statistically significant association between the occurrence of prematurity and the mother's weight gain during pregnancy. Studies by Szablewska A et al., as well as Girard C et al., also described that preterm infants were significantly more likely to be born to mothers who were overweight or obese [14,17]. A study by Barišić A and Finderle A which included 14,799 pregnant women, also showed that mothers with a higher BMI were more likely to give birth to premature infants [18]. In the studies by Gandhi S et al. and Liu P et al., the results showed that underweight mothers gave birth to preterm infants statistically significantly more often, which was not observed in our research [7,8]. We note that in our research, the number of underweight mothers was significantly lower compared to the aforementioned studies. In their study, Santos S et al. described that mothers with inadequate and excessive GWG gave birth to premature infants more often, as did underweight mothers and mothers with high BMI [19]. Yan Q et al. reported that mothers with low GWG statistically significantly more often gave birth to premature infants [20].

Newborns of mothers with high BMI in our study had statistically significantly higher birth weight, which is consistent with the study by Gandhi S et al. [7]. Our research also showed that maternal GWG during pregnancy was associated with the birth weight of newborns. Similar results were reported by Liu X et al., Nepal J et al., Gearhart R et al, and Yan Q et al. [9,10,16,20]. Santos S et al. also described that overweight and obese mothers were more likely to give birth to LGA infants, but also that these mothers had a lower risk of giving birth to small for gestational age (SGA) babies [19]. Barišić A and Finderle A described that pregnant women with high BMI statistically significantly more often gave birth to infants with low birth weight, but also to macrosomic infants [18]. Liu P et al. described that malnourished mothers gave birth to children with low birth weight significantly more often [8]. Yan Q et al. showed that mothers with low GWG were more likely to give birth to babies with low birth weight [20].

The study by Barišić A and Finderle A verified that mothers with high BMI at the beginning of pregnancy were more likely to give birth to babies with lower Apgar scores, which we also demonstrated (χ² = 34.10; p < 0.001) [18]. Zhu T et al. and Victor A et al. also described lower Apgar scores in significant correlation with high BMI [21,22]. In our study, we recorded lower Apgar scores in newborns of mothers with excessive GWG compared to other categories. The same observations were reported by Goławski K et al. [23].

The results of our study showed that infants of mothers with higher BMI were hospitalized in the NICU more often. These results are in line with those of the study by Gandhi S et al. [7], which also described that infants of mothers with higher BMI required hospitalization in the NICU more often. Our results are also consistent with those of the study by Liu P et al. [8]. The study by Eltayeb RA and Khalifa AA did not demonstrate a relationship between maternal BMI and the need for NICU hospitalization [24]. Our study did not find a significant association between the categories of maternal GWG and the need for hospitalization of the newborn in the NICU. In contrast, the study by Gearhart R et al. presented a statistically significant association between both excessive and insufficient maternal GWG and the need for hospitalization of the baby in the NICU [16].

Our study found a statistically significant association between maternal BMI and maternal GWG and the presence of diseases or complications during pregnancy, including gestational hypertension, gestational diabetes mellitus, preeclampsia, postpartum hemorrhage, etc. The incidence of disease was higher in groups of overweight and obese mothers, as well as in mothers with high GWG. The study by Zhang Y et al. also demonstrated that pregnancy complications, such as preeclampsia, eclampsia, hypertension, and gestational diabetes, are more common in women with high BMI [13]. Similar results were also presented in the studies by Gandhi S et al., Yan Q et al., Hung TH et al., and Yang Z et al. [7,20,25,26]. Santos S et al., in a large meta-analysis of 265,270 births, demonstrated that maternal health-related complications during pregnancy were statistically significantly more common in mothers with high BMI at the beginning of pregnancy, as well as in mothers with high GWG [19].

We additionally analyzed the association between the frequency of IVF pregnancies and maternal BMI categories. The analysis showed that IVF pregnancies were statistically significantly more frequent in overweight and obese mothers. The study by Dayan N et al. did not describe a statistically significant difference between the BMI categories of mothers who conceived by IVF [27].

This study has limitations, as it was unicentric, conducted during one calendar year, and performed at a tertiary referral center, which could lead to biased effect estimates. Additionally, there may be measurement errors in self-reported weight values and heterogeneity of the composite outcome. The study can serve as the basis for further monitoring and planning of a longer, prospective, multicentric, and more detailed study.

## Conclusions

The conducted research provides data on the statistically significant and non-negligible association of inadequate maternal BMI before conception and non-optimal GWG during pregnancy with adverse perinatal outcomes. A statistically significant association was found between maternal BMI before pregnancy and type of delivery, prematurity, birth weight, Apgar score, need for NICU hospitalization, presence of maternal disease, and mode of conception, while GWG during pregnancy was significantly associated with newborn birth weight, Apgar score, and presence of maternal disease.

Preconception counseling and preventive promotion of body weight control must become a priority in public health. Women, especially future mothers, should be motivated to adopt a healthy lifestyle before and during pregnancy in order to reduce the prevalence of high BMI among pregnant women. Therefore, it is necessary to emphasize the importance of education and raising awareness about adequate nutrition, physical activity, normal body weight, and regular check-ups. This will consequently have positive effects on reducing the risk of less favorable perinatal outcomes for the mother and newborn.
